# Trained immunity induced by DAMPs and LAMPs in chronic inflammatory diseases

**DOI:** 10.1038/s12276-025-01542-w

**Published:** 2025-10-01

**Authors:** Hee Young Kim, Won-Woo Lee

**Affiliations:** 1 Department of Microbiology and Immunology, Seoul National University College of Medicine, Seoul, Republic of Korea; 2 Institute of Endemic Diseases, Seoul National University Medical Research Center, Seoul, Republic of Korea; 3 Laboratory of Autoimmunity and Inflammation, Department of Biomedical Sciences, Seoul National University College of Medicine, Seoul, Republic of Korea; 4 Seoul National University Cancer Research Institute; Ischemic/Hypoxic Disease Institute, Seoul National University Medical Research Center, Seoul National University Hospital Biomedical Research Institute, Seoul, Republic of Korea

**Keywords:** Innate immunity, Inflammation

## Abstract

The immune system has traditionally been divided into innate and adaptive branches, with immunological memory considered a hallmark of adaptive immunity. However, recent studies reveal that innate immune cells can also exhibit memory-like properties, known as trained immunity. This phenomenon involves the long-term functional reprogramming of innate immune cells following exposure to exogenous or endogenous stimuli, mediated by epigenetic and metabolic changes. Trained immunity enhances responses to subsequent unrelated challenges and serves as a protective mechanism against reinfection. Nonetheless, it may also contribute to the development of chronic inflammatory diseases such as autoimmune disorders, allergies and atherosclerosis. Whereas much of the research has focused on pathogen-associated molecular patterns as inducers of trained immunity, emerging evidence highlights that sterile inflammation, driven by damage-associated molecular patterns and lifestyle-associated molecular patterns, can similarly induce this immune adaptation. Here we examine the molecular mechanisms underlying damage-associated molecular pattern- and lifestyle-associated molecular pattern-induced trained immunity and their roles in chronic inflammation. This Review also discusses central trained immunity, characterized by the durable reprogramming of hematopoietic stem and progenitor cells, and its implications in disease progression. Finally, potential therapeutic strategies targeting metabolic and epigenetic pathways are considered. Understanding noninfectious stimuli-induced trained immunity offers new insights into chronic inflammatory disease management.

## trained immunity reveals innate cells can remember challenges

The immune system has two parts: innate and adaptive immunity. Scientists used to think only adaptive immunity had memory, but recent studies show innate immunity can also remember. Trained immunity involves long-term changes in innate immune cells, such as macrophages, due to vaccines or other stimuli, leading to better responses to future infections. Researchers have found that not just infections but also noninfectious factors such as diet and stress can trigger trained immunity. This Review reviews recent findings on how certain molecules, called damage-associated molecular patterns and lifestyle-associated molecular patterns, can induce trained immunity. These molecules are released from damaged cells or accumulated owing to poor clearance and can cause chronic inflammation, contributing to diseases such as atherosclerosis and chronic kidney disease. The Review concludes that understanding trained immunity could lead to new treatments for chronic inflammatory diseases by targeting these pathways.

This summary was initially drafted using artificial intelligence, then revised and fact-checked by the author.

## Introduction

The immune system is traditionally divided into innate and adaptive immunity, distinguished by specificity and memory. Innate immunity, present in all cellular organisms, offers a rapid, nonspecific defense against pathogens. By contrast, adaptive immunity, which evolved in vertebrates, provides a slower, specific response and develops long-term memory to improve defense against previously encountered pathogens. This understanding led to the incorrect belief that adaptive immunity was the sole mechanism of immunological memory^1^.

From an evolutionary standpoint, immunity encompasses an organism’s ability to resist or combat diseases, pathogens and harmful agents, a concept of considerable significance in evolutionary biology, as it informs the adaptive strategies organisms use in their environments^2,3^. Immunological memory is recognized as a crucial evolutionary trait that enhances host survival upon reinfection^2^. Therefore, it is unlikely that a characteristic as critical as immunological memory is exclusive to adaptive immunity. Further, this system has developed only in vertebrates, which account for merely ~1–2% of living species, whereas innate immune responses are widespread across the broader spectrum of life^3^.

Studies in plant immunology have revealed that plant host defenses exhibit adaptive characteristics, such as systemic acquired resistance^4^. Furthermore, evidence of adaptive features within innate immune responses has been documented in various invertebrate lineages^5^, challenging the notion that immunological memory is confined to adaptive immunity^3^.

In humans, live attenuated vaccines such as Bacillus Calmette–Guérin (BCG) offer cross-protection against unrelated pathogens in infants^6,7^, independent of T and B cells, and primarily via macrophages^8,9^. In 2011, Netea et al. coined the term ‘trained immunity’ to describe nonspecific protection induced by such vaccines, which involves long-term functional reprogramming of innate immune cells through epigenetic and metabolic changes, distinct from classical epitope-specific adaptive immunological memory, which relies on antigen–receptor interactions^10–13^. Currently, trained immunity is defined as the long-term functional reprogramming of innate immune cells, induced by either exogenous or endogenous insults, resulting in enhanced effector function upon unrelated secondary stimulation after returning to an inactive state^14^.

Trained immunity has evolved as a beneficial mechanism for nonspecific protection against future infections but may also contribute to maladaptive outcomes, including autoimmune diseases, allergies and chronic inflammation, through amplified immune responses^14,15^. Although much research has focused on the triggering of trained immunity by microbial insults, such as pathogen-associated molecular patterns (PAMPs), it is plausible that this process can also be triggered by sterile inflammatory insults, which play a critical role in the pathogenesis of various inflammatory diseases in humans. Sterile (noninfectious) inflammation is classically initiated by the recognition of damage-associated molecular patterns (DAMPs), which are endogenous molecules released from damaged or dying cells, including ATP, high mobility group box 1 (HMGB1) and heme. These molecules are capable of activating innate immune responses. More recently, lifestyle-associated molecular patterns (LAMPs) such as oxidized low-density lipoprotein (oxLDL), cholesterol crystals and monosodium urate, have been proposed as a trigger of sterile inflammation^16^. LAMPs are immunostimulatory molecules not derived from pathogens or cellular injury but rather from lifestyle-related factors and chronic conditions. LAMPs are often inefficiently cleared by the immune system, potentially impairing the resolution of inflammation and contributing to the development of chronic noncommunicable diseases.

Recent research has revealed that sterile inflammatory factors such as oxLDL and components of the Western diet (WD) also induce trained immunity in human monocytes^17–20^. These factors may contribute to chronic inflammatory conditions and enhance trained immunity in macrophages and monocytes^21^.

Whereas the basic concepts, inducers and mechanisms of trained immunity triggered by various microbial insults have been extensively reviewed, the role of DAMPs in this context has received comparatively limited attention. Our review provides several important updates and novel perspectives that differentiate it from earlier publications. Specifically, we present an up-to-date overview of findings from 2023 to 2025 on how endogenous sterile danger signals, such as uremic toxins (for example, indoxyl sulfate (IS)), heme and proinflammatory cytokines, can induce trained immunity in both circulating myeloid cells and hematopoietic progenitors. These recent discoveries shed light on the underlying mechanisms, including epigenetic remodeling, immunometabolic reprogramming and transcriptional changes that underpin the persistence of innate immune memory and its role in chronic inflammation. Furthermore, our review places particular emphasis on the clinical implications of DAMP-induced trained immunity by linking these mechanistic insights to the pathogenesis of chronic inflammatory and metabolic diseases, such as chronic kidney disease (CKD), atherosclerosis, rheumatic diseases and aging. This disease-oriented perspective has not been comprehensively covered in prior reviews. Importantly, we also discuss the emerging concept of central trained immunity, which involves long-term reprogramming at the level of hematopoietic stem and progenitor cells (HSPCs). This is a recently identified mechanism that was not included in earlier reviews.

Collectively, this review aims to reposition DAMPs and related sterile danger signals, including LAMPs, as key noninfectious inducers of trained immunity. We propose trained immunity as a unifying framework that bridges innate immune memory with the development and persistence of chronic inflammation, while also exploring potential therapeutic implications.

## General concept of trained immunity

**Fig. 1:**
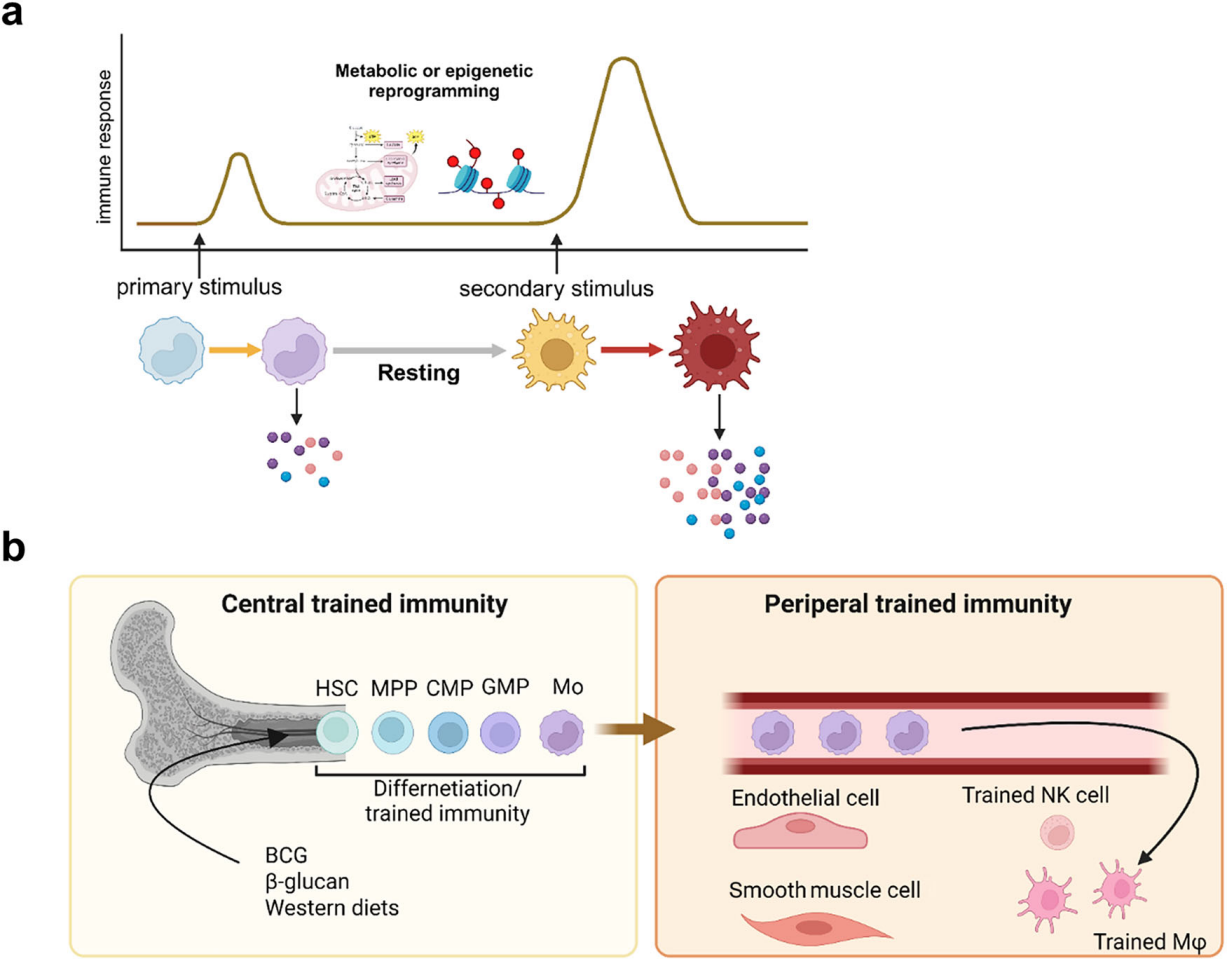
Definition and classification of trained immunity. **a**, Trained immunity is defined as innate immune memory, characterized by an enhanced production of proinflammatory cytokines or chemokines upon secondary stimulation that is distinct from the initial stimulus. Trained immunity can persist for days to weeks in innate immune cells (for example, monocytes, NK cells and tissue-resident macrophages) and non-immune cells (for example, ECs and SMCs) following exposure to inducers such as PAMPs or DAMPs, a phenomenon referred to as peripheral trained immunity. **b**, Trained immunity can also persist for months or even years through the reprogramming of HSPCs in the bone marrow, which is referred to as central trained immunity. The figure was created using BioRender.com. CMP, common myeloid progenitor; MPP, multipotent progenitor; Mo, monocytes; Mφ, macrophages.

The adaptation of the immune system to internal or external stimuli can be classified into four distinct functional states: differentiation, priming, tolerance and trained immunity. Differentiation involves long-term changes in innate immune cells, leading to lineage commitment or specialized functions. Priming enhances immune responsiveness after an initial stimulus, resulting in a sustained activated state without returning to baseline. Tolerance entails diminished responsiveness to subsequent stimuli, serving to prevent excessive inflammation. By contrast, trained immunity entails a transient activation of innate immune cells that returns to baseline after the initial stimulus, while epigenetic reprogramming persists. Upon reexposure, this results in an enhanced secondary response, resembling memory-like behavior in lymphocytes^14,22^ (Fig. 1a). Unlike priming, trained immunity is marked by a return to a resting state before reactivation^22^. Initial stimuli of trained immunity include microbial agents such as bacterial and fungal agents (for example, β-glucan), vaccines such as BCG and endogenous signals such as oxLDL. Early research focused on peripheral blood cells, natural killer (NK) cells, innate lymphoid cells and tissue macrophages^11,23–25^, primarily examining a duration of days or weeks (termed peripheral trained immunity). However, studies have expanded to explore long-term reprogramming in bone marrow progenitors, which is known as central trained immunity^26^ (Fig. 1b). For instance, β-glucan and BCG trigger the reprogramming of hematopoietic stem cells (HSCs) in the bone marrow, leading to myeloid progenitor proliferation^26,27^. In murine models, β-glucan-induced trained immunity in HSCs has been linked to interleukin (IL)-1β and GM-CSF signaling pathways. Furthermore, BCG-induced trained immunity results in epigenetically reprogrammed macrophages, which confer protection against virulent *Mycobacterium tuberculosis* infections. Recent studies have demonstrated that in humans, BCG vaccination alters gene expression and epigenetic chromatin accessibility mediated by IL-1β in HSCs, facilitating the differentiation of myeloid progenitors^28^.

The regulation of trained immunity involves several critical mechanisms, including epigenetic reprogramming, metabolic reconfiguration and changes in gene expression. A fundamental aspect of trained immunity is epigenetic reprogramming, which encompasses alterations in the epigenetic landscape of immune cells. This reprogramming alters histone modifications, such as H3K4me1, H3K4me3 and H3K27ac at promoters and enhancers, as well as DNA methylation patterns in genes associated with trained immunity, including those encoding inflammatory cytokines and transcription factors^11^.

H3K4me3 and H3K27ac are well-established histone markers associated with trained immunity. Numerous studies indicate that transcriptional regulation in trained immunity is mediated by the accumulation of H3K4me3 at the promoters of target genes^12,17^. Fanucchi et al. revealed that β-glucan-induced trained immunity is regulated by long noncoding RNAs, such as UMLILO. These function through the WD-repeat-containing protein 5 (WDR5)-mixed lineage leukemia protein 1 (MLL1) complex to promote the H3K4me3 enrichment of target genes, including chemokines such as Cxcl. Thus, this highlights an additional layer of epigenetic control^29^.

Moreover, metabolic alterations are crucial in mediating trained immunity^13^. Initial exposures prompt shifts in cellular metabolism that influence how immune cells generate and utilize energy, thereby enhancing their functionality during subsequent pathogen encounters. For example, β-glucan promotes the expression of glycolysis-related genes via the mammalian target of rapamycin (mTOR)–hypoxia-inducible factor 1 alpha (HIF-1α) pathway, facilitating a transition from oxidative phosphorylation (OXPHOS) to aerobic glycolysis^13^. In addition, pathways involving glutaminolysis and cholesterol synthesis further contribute to β-glucan-mediated trained immunity, underscoring the importance of metabolic reprogramming for an augmented immune response^30,31^. The accumulation of fumarate inhibits KDM5 histone demethylases, resulting in the retention of H3K4me3, a hallmark of trained immunity. Similarly, mevalonate, an intermediate in cholesterol synthesis, enhances histone modifications by activating IGF-1R and mTOR signaling pathways^30,31^. BCG-induced trained immunity also involves glycolysis-driven epigenetic modifications^12^. Likewise, sterile inflammatory molecules such as oxLDL induce epigenetic changes through metabolic reprogramming^32^. Furthermore, β-glucan-induced Set7 methyltransferase modulates the plasticity of OXPHOS by catalyzing histone methylation at genes such as MDH2 and SDHB^33^. These findings highlight the critical interplay between metabolic reconfiguration and epigenetic reprogramming in sustaining long-term immune responses.

Targeting of trained immunity offers therapeutic potential for inflammatory and autoimmune diseases. Vaccines such as BCG and PAMPs can provide protection against infections, such as severe acute respiratory syndrome coronavirus 2 (SARS-CoV-2)^34^, sepsis^35^ and certain cancers^36^, while endogenous triggers such as oxLDL and uremic toxins may drive maladaptive trained immunity, contributing to chronic conditions such as cardiovascular and kidney diseases^37^. Modulating trained immunity pathways could offer new strategies to control immune responses and manage chronic inflammatory diseases.

## Triggering of trained immunity by DAMPs and LAMPs

**Fig. 2:**
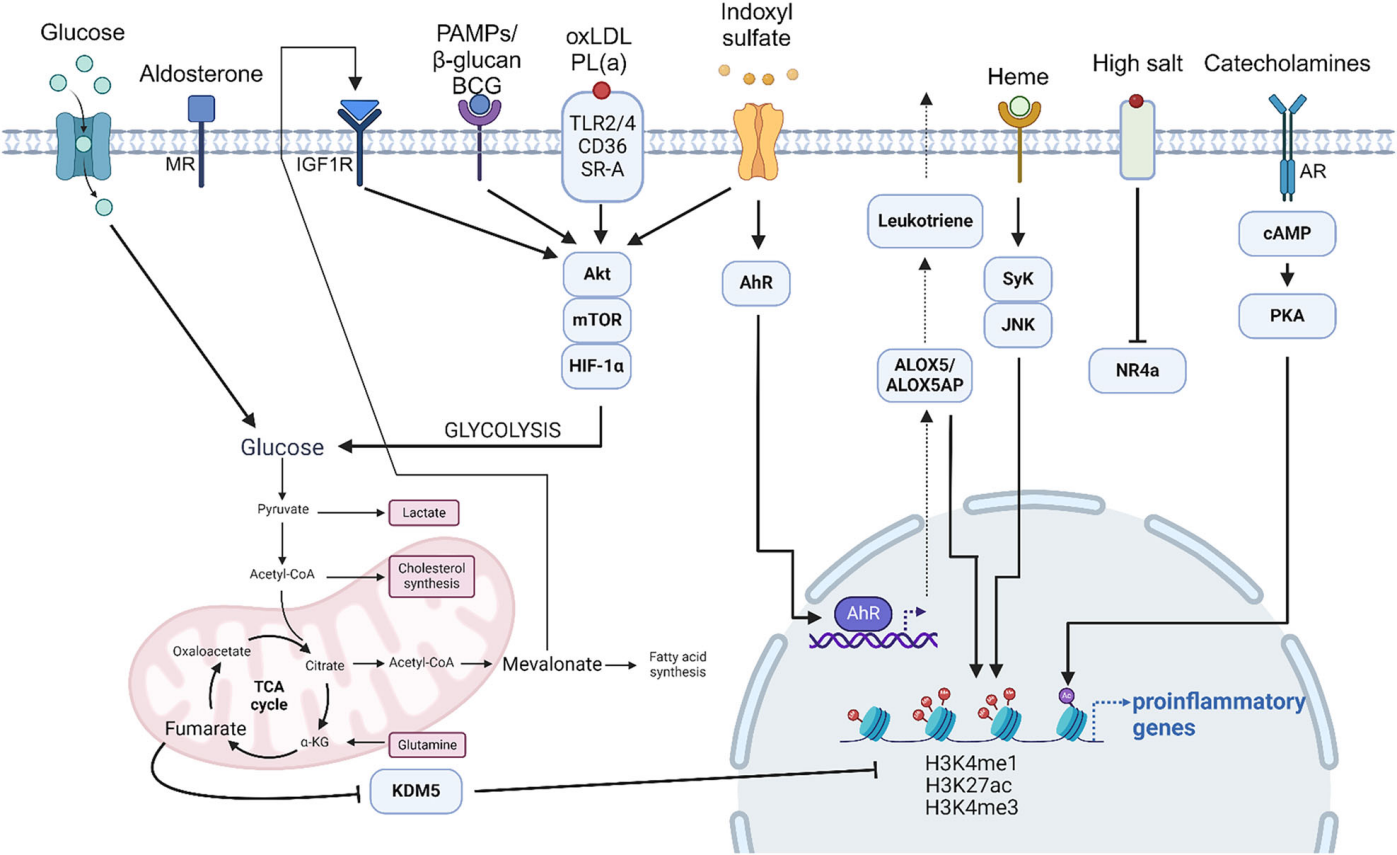
Schematic diagram of the mechanisms of trained immunity induced by several sterile danger signals. The major mechanisms underlying trained immunity involve metabolic and epigenetic reprogramming. Trained immunity induced by PAMPs (for example, β-glucan, BCG) enhances glycolysis through mTOR–HIF-1α signaling and initiates epigenetic modifications via markers such as H3K4me1, H3K4me3, H3K27ac or DNA methylation. Recent studies have demonstrated that several endogenous molecules (for example, oxLDL, PL(a), IS, heme), as well as the WD, can induce trained immunity. oxLDL elevates glycolysis and OXPHOS via mTOR–HIF-1α signaling, similar to PAMPs, while promoting epigenetic reprogramming through histone modifications. IS enhances glycolysis and OXPHOS through mTOR signaling and induces epigenetic reprogramming via the AhR–AA pathway. Heme drives trained immunity through Syk/JNK signaling. In addition, catecholamines and aldosterone induce trained immunity through the β-adrenergic receptor–cAMP–PKA pathway and the MR pathway, respectively. Hyperglycemia and high-salt diets modulate metabolic reprogramming, with hyperglycemia contributing to metabolic shifts and high salt downregulating NR4a. PL(a), Lipoprotein (a). The figure was created using BioRender.com.

Historically, the immune response was seen as a series of processes aimed at protecting the host from external threats by distinguishing self from nonself. In this context, the innate immune system initiates inflammation by recognizing PAMPs through pattern recognition receptors (PRRs) to defend against foreign pathogens. However, it is now clear that inflammatory responses can occur without infections, a phenomenon called sterile inflammation induced by DAMPs and LAMPs, indicating the presence of molecules besides PAMPs that can trigger inflammation^38^. Most research on trained immunity has predominantly focused on microbial triggers; however, emerging evidence suggests that DAMPs and LAMPs also play important roles in this process. Given that PAMPs, DAMPs and LAMPs are all recognized by similar PRR systems and contribute to inflammatory responses, it is plausible that nonmicrobial stimuli can similarly induce trained immunity. The identification of oxLDL as a noninfectious inducer of trained immunity in monocytes led Crisan et al. to propose the concept of DAMP-induced trained immunity^39^. Recent evidence has shown that DAMPs, including uric acid, heme and uremic toxins, can promote trained immunity. Moreover, trained immunity can be induced by other sterile danger signals, such as hormones such as aldosterone, and LAMPs such as a WD and high-salt intake (Fig. 2). These findings broaden the scope of trained immunity beyond infectious triggers, highlighting the role of sterile inflammatory signals in the long-term reprogramming of innate immune responses.

### oxLDL

Atherosclerosis, the leading cause of cardiovascular disease (CVD), is marked by chronic inflammation within the arterial walls, driven by both innate and adaptive immune responses^40^. Monocytes and macrophages are crucial in forming and rupturing lipid-rich plaques in this process^41^. HAECs and coronary smooth muscle cells (SMCs), which express PRRs such as Toll-like receptors (TLR)2 and TLR4, also contribute to the development of atherosclerosis^42,43^. oxLDL, a modified form of LDL, plays a central role in atherosclerosis pathogenesis. When macrophages take up oxLDL, foam cells form, contributing to plaque buildup that narrows and hardens arteries, increasing the risk of myocardial infarction or cerebrovascular accidents.

In 2014, Bekkering demonstrated that oxLDL induces trained immunity in monocytes^17^. Preexposure to oxLDL triggers a long-lasting proinflammatory and proatherogenic phenotype in monocytes, marked by heightened responses to TLR2 and TLR4 agonists and increased foam-cell formation. oxLDL-trained monocytes show an upregulation of CD36 and scavenger receptor A, key scavenger receptors for oxLDL, while downregulating cholesterol efflux transporters ABCA1 and ABCG1. Epigenetic reprogramming induced by oxLDL includes trimethylation of histone 3 (H3K4me3) at proinflammatory gene promoters, leading to an increased production of cytokines^17^. Moreover, oxLDL promotes metabolic reprogramming, enhancing glycolysis and oxygen consumption through the upregulation of glycolytic enzymes PFKFB3 and PFKP^32^. Omics profiling of oxLDL-trained macrophages highlights mitochondrial-linked metabolic changes, including increased mitochondrial reactive oxygen species (mitoROS), biogenesis and OXPHOS^44^. The pharmacological inhibition of glycolytic or tricarboxylic acid (TCA) cycle pathways reduces oxLDL-induced trained immunity, whereas higher glucose levels worsen it^32,44^.

The signaling pathways involved in oxLDL-induced trained immunity are linked to scavenger receptor CD36 and PRRs such as TLR2 and TLR4, which promote foam-cell formation and cytokine production. OxLDL also activates mTOR, generating ROS and stabilizing HIF-1α, leading to the transcription of its target genes^42–44^. Similar to PAMP-induced trained immunity, the enduring effects of oxLDL exposure are mediated through these pathways.

### WD

In modern Western societies, over 80% of mortality is linked to noncommunicable diseases, many influenced by aging and the consumption of calorically dense Western-type diets. These diets, high in fat and sugar, are associated with conditions such as type II diabetes, obesity and CVDs.

Using an *Ldlr*^−/−^mouse model of atherosclerosis, Christ et al. demonstrated that prolonged WD consumption induces long-lasting trained immunity in myeloid cells. In *Ldlr*^−/−^ mice, WD feeding causes systemic inflammation and reprogramming of myeloid cells, marked by increased myelopoiesis and transcriptional changes in myeloid precursor cells such as granulocyte–monocyte progenitors (GMPs). WD-induced inflammation subsides with a normal chow diet, but myeloid cell responses remain heightened owing to epigenetic reprogramming^19^.

Transcriptional and epigenetic analyses of GMPs revealed that IL-1β-mediated signaling is key in WD-induced trained immunity. Administering recombinant IL-1 receptor antagonist (IL-1ra) reduced systemic inflammation in WD-fed mice and suppressed oxLDL-induced trained immunity in vitro. Previous research showed oxLDL activates the NLRP3 inflammasome, a pathway linked to atherosclerosis. In *Nlrp3*^−/−^/*Ldlr*^−/−^ mice, WD-induced inflammation, myeloid progenitor proliferation and cell reprogramming were completely blocked^19^. These findings highlight NLRP3’s role in trained immunity following WD exposure and its contribution to chronic inflammatory diseases.

Recent studies show that saturated fatty acids, abundant in the WD, induce trained immunity and a hyperinflammatory response to lipopolysaccaride (LPS)^45^. Mice fed a diet enriched in saturated fatty acids (ketogenic diet) show increased free palmitic acid and palmitic-acid-associated lipids, worsening endotoxemia and systemic inflammation via ceramide synthesis in macrophages^45,46^. Early hyperlipidemia in *ApoE*^−/−^ mice on a high-fat diet triggers metabolic reprogramming by *S*-adenosylhomocysteine, increasing acetyl-CoA–cholesterol synthesis and decreasing glycolysis. This results in the upregulation of uremic toxins and metabolites including palmitate that promote trained immunity. Lipid metabolism pathways are critical for regulating immune responses, including trained immunity^27,31,47^. Lipid profiles are shaped by both dietary fat and endogenous cholesterol synthesis. A lipidomic study showed that lipid mediators (LMs) from long-chain polyunsaturated fatty acids and lipoxygenase-derived LMs are essential for BCG-induced trained immunity in human monocytes^48^.

### Hyperglycemia

Diabetes is a noncommunicable disease marked by chronic low-grade inflammation, contributing to various complications such as CVDs. Hyperglycemia, a key risk factor for diabetes, accelerates the progression of atherosclerosis. Elevated glucose activates monocytes and enhances proinflammatory gene expression through oxidative stress and NF-κB^49^. In M1-like proinflammatory macrophages, glucose primarily serves as a glycolysis substrate, whereas M2-like anti-inflammatory macrophages rely on OXPHOS^50^. Research indicates hyperglycemia induces trained immunity in macrophages and precursors, further promoting atherosclerosis^18,51^.

In bone marrow-derived macrophages (BMDM), high glucose (20 nmol/L) causes metabolic reprogramming, boosting glycolytic ATP and lactate production, along with proinflammatory gene expression through epigenetic changes^18^. Bone marrow transplantation from diabetic mice into normoglycemic *Ldlr*^−/−^ mice increases aortic root atherosclerosis, indicating that the trained immunity phenotype persists posttransplant. The transcription factor runt-related transcription factor 1 (Runx1) is key in hyperglycemia-induced trained immunity^18^. Runx1 target genes are enriched in immune cells from type 2 diabetic patients, and Runx1 inhibition in murine BMDM reduces trained immunity. In addition, hyperglycemia-induced trained immunity in human monocytes is regulated by epigenetic changes in the mixed lineage leukemia (MLL) family of genes^51^.

### Uremic toxins

CKD is a growing global health issue. The loss of renal function leads to the accumulation of over 100 uremic toxins, which contribute to cardiovascular risk by inducing oxidative stress and proinflammatory cytokines^52,53^. Consequently, CVD is a leading cause of mortality in patients with end-stage renal disease (ESRD)^54^. IS, a major uremic toxin, is produced from dietary tryptophan fermentation by gut microbiota. Due to poor clearance in hemodialysis, IS accumulates in the serum of patients with CKD^55^.

IS triggers release of proinflammatory cytokines such as tumor necrosis factor (TNF) through aryl hydrocarbon receptor (AhR) signaling in monocytes/macrophages, thereby promoting atherosclerosis^56,57^. Kim et al. demonstrated that IS can induce trained immunity in human monocytes^21^. Further, in vitro IS-trained monocytes exhibit an increased production of proinflammatory cytokines and chemokines, including TNF, IL-6, IL-1β and MCP-1, while displaying reduced levels of the anti-inflammatory cytokine IL-10 upon LPS stimulation. Thus, this suggests that IS serves as an inducer of trained immunity. Moreover, other protein-bound uremic toxins such as *p*-cresyl sulfate and hippuric acid do not affect TNF or IL-6 secretion.

IS promotes glycolysis, OXPHOS, and increases H3K4me3 localization to TNF and IL-6 promoters. Transcriptional profiling shows IS-induced trained immunity is regulated through enhanced 5-lipoxygenase (ALOX5) expression via the AhR-dependent arachidonic acid (AA) pathway. Monocytes from patients with ESRD exhibit increased ALOX5 expression, and after 6 days of ex vivo training, they exhibit heightened TNF and IL-6 production in response to LPS stimulation. Furthermore, TNF and IL-6 production is also enhanced in monocytes from healthy controls when trained with uremic sera from patients with ESRD. Consistently, IS-trained mice and their splenic myeloid cells were found to display increased TNF production following both in vivo and ex vivo LPS stimulation^21^.

A recent study found that trimethylamine *N*-oxide (TMAO), a uremic toxin elevated in ESRD, induces trained immunity in human aortic endothelial cells (HAECs). TMAO triggers the transdifferentiation of HAECs into innate immune-like cells, promoting vascular inflammation. This process involves TMAO-driven metabolic reprogramming, which includes enhanced glycolytic pathways and increased mitoROS production, thereby establishing trained immunity in HAECs through mechanisms that involve endoplasmic reticulum (ER) stress and protein kinase RNA-like ER kinase (PERK) receptor signaling^58^.

### Heme

Heme, an evolutionarily conserved molecule with a tetrapyrrole ring and iron atom, is released during infections such as malaria and sepsis. Extracellular labile heme activates myeloid cells as an alarmin, triggering TNF secretion via the PRR pathway^59,60^. Heme also induces long-term trained immunity, evidenced by increased H3K27ac at TNF and IL-8 promoters and modulated spleen tyrosine kinase (Syk)/c-Jun N-terminal kinases (JNK) activation^61^. Transcription factors Nfix, Runx1 and Nfe2l2, along with Bach2 dissociation, are crucial for heme-induced myelopoiesis and trained immunity. DAMP-induced trained immunity can contribute to disease pathology not only in chronic inflammatory conditions but also in certain infectious diseases. Notably, heme-induced trained immunity has been shown to exacerbate immunopathology in infectious contexts. In sepsis, it may offer early protection against bacterial infection but also lead to harmful outcomes, such as excessive inflammation and tissue damage during endotoxic shock or delayed sepsis^61^. In sickle cell disease, chronic heme exposure drives persistent trained immunity in myeloid cells and progenitors, promoting thromboinflammatory complications through increased tissue factor expression^62^. These findings highlight heme as both an acute danger signal and a long-term modulator of innate immune memory, linking hemolysis to chronic inflammation and immune dysregulation.

### Other triggers

Catecholamines, including dopamine, norepinephrine and epinephrine (adrenaline), are endogenous inflammation inducers and key players in stress responses and chronic inflammatory conditions, including CVD. In vitro studies show that acute exposure to adrenaline or noradrenaline reduces TNF and IL-6 production in LPS-stimulated human monocytes. However, long-term catecholamine exposure enhances TNF and IL-6 production upon restimulation with LPS^63^. This catecholamine training is linked to increased glycolysis and OXPHOS before secondary stimulation. Similar to β-glucan-induced trained immunity, the β-adrenergic receptor–cAMP–PKA signaling pathway is involved in catecholamine-induced trained immunity. In peripheral blood mononuclear cells from patients with pheochromocytoma/paraganglioma, neuroendocrine tumors that release high levels of catecholamines, TNF production is increased, while IL-10 production is reduced upon ex vivo LPS stimulation, compared with that of control cells from patients with essential hypertension. This suggests that catecholamines contribute to trained immunity^63^. Peripheral CD14^+^ monocytes isolated from patients with pheochromocytoma/paraganglioma exhibit upregulated proinflammatory genes and increased H3K4me3 on TNF and IL1B promoters.

Aldosterone, which is linked to hypertension and atherosclerosis^64^, induces trained immunity in human monocytes^65^. Aldosterone or serum from patients with primary hyperaldosteronism stimulates TNF and IL-6 production in response to Pam3cys, via mineralocorticoid receptor (MR) activation. The underlying mechanism for this appears to be epigenetic reprogramming as aldosterone enhances methylation in the promoters of genes involved in fatty acid synthesis (for example, FASN, ACACA and ELOVL6) as well as those of proinflammatory genes such as TNF and IL-6.

Recent research demonstrates that a high-salt diet induces trained immunity in HSPCs by downregulating the expression of the NR4a family and reducing mitochondrial OXPHOS. This trained immunity impairs recovery from hemorrhagic and ischemic strokes, even when diet normalization occurs before stroke onset^66^. Moreover, a high-salt diet boosts macrophage trained immunity, resulting in hyperinflammation during repeated pathogenic exposures. This response is driven by chemokine (C–C motif) ligand 2 (CCL2)–C–C chemokine receptor type 2 (CCR2) signaling and dysregulated mTORC1 activation in a mouse model of CKD. In addition, transplanting monocytes from CKD mice on a high-salt diet worsens renal inflammation^67^ (Table 1).

**Table 1:** Sterile inflammatory inducer of trained immunity involved in various chronic inflammatory diseases.

Inducer	Disease	Receptor	Mechanism	Reference
oxLDL	CVD	TLR2, 4 CD36 Scavenger receptor A	mTOR–AKT–HIF-1α Metabolic rewiring (glycolysis, OXPHOS) Epigenetic reprogramming ROS	^17,32,42–44^
WDs	CVD	NLRP3	HSC myeloid precursor Epigenetic reprogramming IL-1β signaling	^19^
Hyperglycemia	Diabetes CVD	HK	Metabolic rewiring Epigenetic modification MLL family RUNX1	^18,49^
IS	CKD CVD	AhR	AA pathway Metabolic rewiring (glycolysis, OXPHOS) Epigenetic reprogramming	^21^
TMAO	CKD CVD	PERK receptor	ER stress (PERK receptor) Metabolic reprogramming (glycolysis, mitoROS)	^58^
Heme	Myelopoiesis	TLR4	Epigenetic reprogramming Syk/JNK	^61^
Catecholamine	CVD	β-adrenergic receptor	Adrenergic receptor–cAMP–PKA signaling mTOR Metabolic rewiring (glycolysis, OXPHOS)	^63^
Aldosterone	Primary hyperaldosteronism	MR	Fatty acid pathway Epigenetic modification (H3K4me3)	^65^
High salt	Stroke CKD	NR4a1	CCL2–CCR2 signaling mTORC1 pathway	^67^

ROS, reactive oxygen species.

IL-4, long considered anti-inflammatory and immunosuppressive, has recently been shown to induce trained immunity under certain inflammatory conditions. Schrijver et al. found that the targeted delivery of IL-4 to myeloid cells in a murine model of sepsis reversed immunoparalysis by reprogramming monocytes into a functionally enhanced state. These cells displayed hallmark features of trained immunity, including restored proinflammatory cytokine responses, increased glycolytic activity and active histone modifications^68^. This challenges the traditional view of IL-4 and broadens the scope of trained immunity to include sterile, nonmicrobial stimuli that reprogram innate immune cells in a context-dependent manner.

## Pathogenic role of trained immunity in chronic inflammatory diseases

Trained immunity was initially observed in mature myeloid cells, but their short lifespan (typically only a few days) raised doubts about how their effects could last for months or years. This paradox was addressed by discovering that trained immunity can also be established at the level of long-lived hematopoietic progenitor cells in the bone marrow (‘central trained immunity’), not just in mature myeloid cells (‘peripheral trained immunity’). This leads to the sustained production of myeloid cells with a proinflammatory phenotype. This persistent reprogramming contributes to the development and maintenance of chronic inflammatory states, thereby playing a key pathogenic role in a range of chronic inflammatory diseases.

### ASCVD

**Fig. 3:**
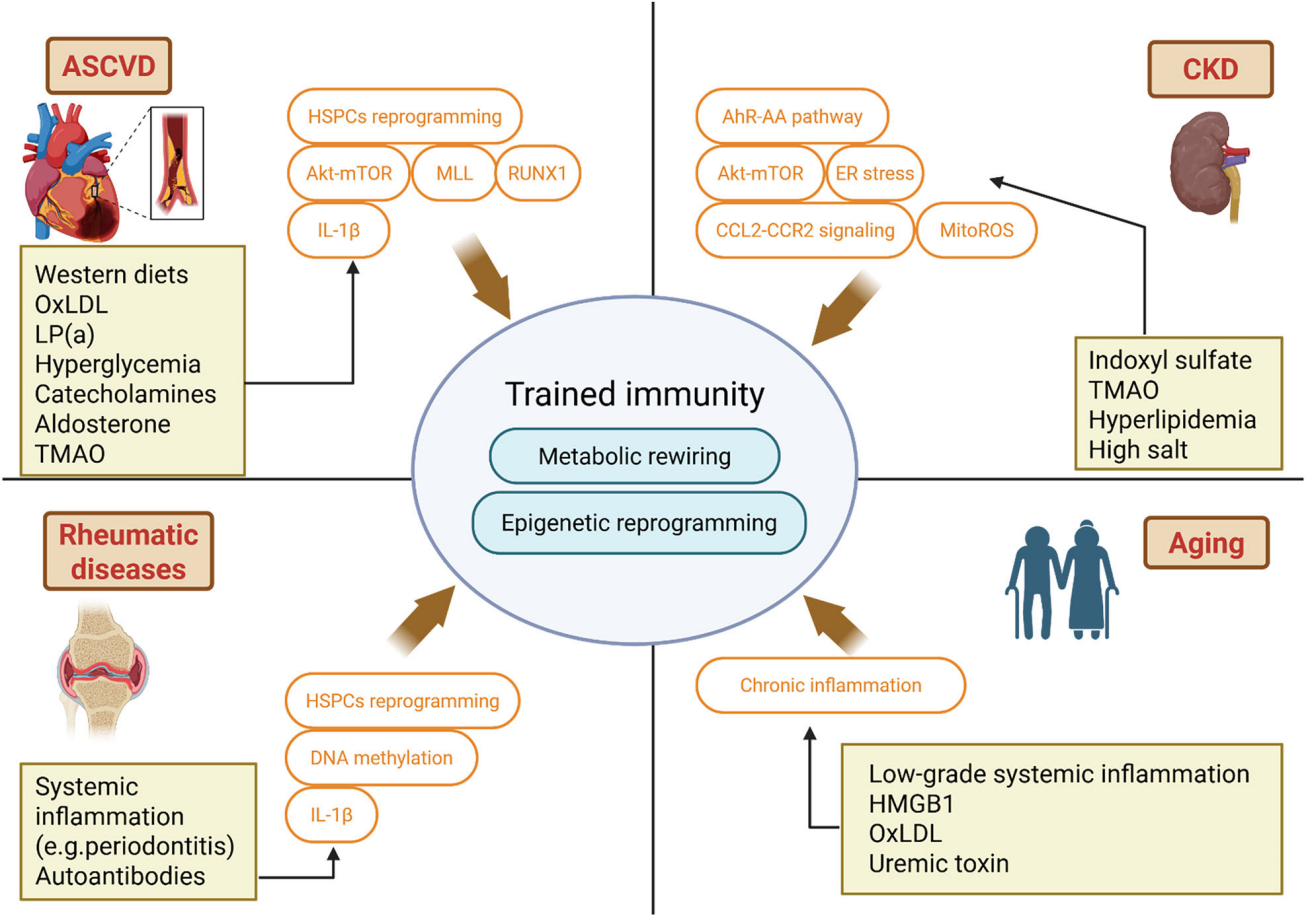
Trained immunity and chronic diseases. Trained immunity plays a pivotal role in the pathogenesis of several chronic diseases, including ASCVD, CKD, rheumatic diseases and age-related conditions. In ASCVD, various endogenous danger signals, such as WDs, oxLDL, LP(a), hyperglycemia, catecholamines, aldosterone and TMAO, induce trained immunity through the reprogramming of HSPCs and the activation of pathways such as Akt–mTOR, MLL family, RUNX1 and IL-1β. This reprogramming sustains inflammatory responses, contributing to the long-term progression of ASCVD. In CKD, uremic toxins (for example, IS and TMAO), hyperlipidemia and high salt levels activate innate immune cells and even non-immune cells (such as ECs), promoting chronic inflammation and progressive renal damage. Similarly, in rheumatic diseases, including RA, gout and SLE, persistent stimulation by endogenous DAMPs, such as autoantibodies, promotes chronic inflammation through trained immunity. In aging, or immunosenescence, there is a decline in adaptive immunity accompanied by a chronic low-grade systemic inflammatory state, termed inflammaging. Over time, trained immunity in response to sustained DAMPs and chronic inflammatory molecules can amplify this inflammation, increasing susceptibility to chronic inflammatory diseases associated with aging. These mechanisms highlight trained immunity as a critical factor in the development and persistence of inflammation-driven diseases across various organ systems and over the lifespan. The figure was created using BioRender.com.

CVD is a well-researched area with respect to the detrimental effects of trained immunity^18,69^. Recent studies underscore the role of trained immunity in circulating innate immune cells, HSPCs and endothelial cells (ECs) in the progression of atherosclerotic CVD (ASCVD). Monocytes and macrophages are particularly critical in the formation of unstable atherosclerotic plaques^70^. Moreover, traditional atherosclerosis risk factors, such as oxLDL, acetylated LDL, lipoprotein(a) (LP(a)), catecholamines and aldosterone, induce trained immunity in human monocytes through metabolic and/or epigenetic reprogramming^17,20,63,65^ (Fig. 3).

ASCVD often coexists with chronic inflammatory diseases including diabetes, CKD and rheumatic diseases. Studies show that conditions such as hyperglycemia in diabetes and uremic toxins such as IS in CKD contribute to trained immunity^18,21,71^. Specifically, hyperglycemia causes metabolic and epigenetic changes via the MLL pathway in BMDMs, while the uremic toxin IS induces metabolic reprogramming and epigenetic modifications via the AhR pathway^21^.

ECs play a key role in atherosclerosis initiation and progression^41^. Activated ECs express damage-associated molecular pattern (DAMP) receptors, MHC molecules, adhesion molecules, cytokines and chemokines. Sohrabi et al. demonstrated that oxLDL induces trained immunity in HAECs, increasing proinflammatory cytokines such as IL-6, IL-8 and MCP-1 upon restimulation with Pam3cys^43^. This trained immunity in HAECs involves metabolic and epigenetic reprogramming, including the activation of the mTOR–HIF-1α signaling pathway. TMAO, produced by the aorta and liver, also induces trained immunity in ECs via metabolic reprogramming^58^. A recent study showed that oxLDL induces a proinflammatory effect in human coronary SMCs via the mTOR–HIF-1α axis, regulated by metabolic and epigenetic modifications^42^.

Both innate immune and non-immune cells can exhibit persistent proinflammatory responses after initial stimulation, driven by metabolic reprogramming and epigenetic modifications such as histone changes, collectively referred to as ‘epigenetic memory’. Despite shared underlying mechanisms, the functional outcomes differ between these cell types. In innate immune cells, trained immunity enhances cytokine production upon secondary challenge, offering protective benefits against infection but potentially contributing to chronic inflammation. By contrast, non-immune cells such as SMCs and ECs, while also responsive to DAMPs and PAMPs via PRRs, exhibit maladaptive phenotypic shifts. These include increased proliferation, migration, extracellular matrix production and endothelial-to-mesenchymal transition, all of which contribute to the progression of vascular pathologies such as atherosclerosis, fibrosis and restenosis^43,58,72,73^.

Recent studies have further demonstrated that both metabolic and systemic inflammatory stress can induce central trained immunity by reprogramming HSPCs. For instance, in WD-fed mice, metabolic stress promotes myeloid-biased differentiation of HSPCs, leading to persistent vascular inflammation^19^. Similarly, systemic inflammatory conditions such as heart failure and stroke have been shown to induce a long-term reprogramming of HSPCs, enhancing the generation of proinflammatory macrophages and contributing to vascular pathology^74,75^.

### CKD

CKD, including ESRD, is closely linked to systemic inflammation due to immune dysfunction^14^. Uremia, common in renal failure, is associated with CVD in patients with CKD^76^. Oxidative stress plays a key role in forming advanced glycation end-products and oxLDL, which trigger inflammation in innate immune cells^77^. OxLDL has been shown to induce trained immunity, thereby promoting ASCVD development. Our findings indicate that IS, a major uremic toxin, induces trained immunity through the AhR-dependent AA pathway, along with metabolic and epigenetic reprogramming^21^. Moreover, monocytes from patients with ESRD exhibit increased TNF and IL-6 production upon ex vivo restimulation with LPS. In vivo studies show that mice exposed to IS and their splenic myeloid cells produce more TNF after LPS stimulation. In addition, hyperlipidemia in a CKD mouse model accelerates atherosclerosis progression by promoting trained immunity^73^. Further, the involvement of LMs also occurs in BCG-induced trained immunity highlights their role, similar to the AA pathway in human monocytes^21^.

The interaction between hyperlipidemia and CKD exacerbates vascular inflammation through trained immunity. In a CKD mouse model with 5/6 nephrectomy and a high-fat diet, increased cytosolic LPS levels activate caspase 11 (caspase 5 in humans) and upregulate trained immunity-related genes in the aorta^73^. Notably, caspase-11 deficiency in this model reduces aortic neointima hyperplasia, inflammatory cell recruitment, N-terminal gasdermin D (N-GSDMD) expression on ECs and aortic IL-1β levels. Thus, this further highlights the role of trained immunity in vascular inflammation^73^.

While direct evidence for central trained immunity in CKD remains limited, chronic inflammatory signaling and uremic milieu may plausibly reprogram HSPCs. Recent work in heart failure models supports this possibility. Specifically, HSPCs in post-heart-failure mice showed transforminggrowth factor-β (TGF-β) pathway suppression, and transplantation of these HSCs was sufficient to induce spontaneous cardiac and renal damage, implicating long-term central immune imprinting^75^.

### Rheumatic diseases

Rheumatic diseases, such as rheumatoid arthritis (RA), systemic lupus erythematosus (SLE), systemic sclerosis and gout, are autoimmune and autoinflammatory disorders marked by chronic inflammation often affecting musculoskeletal and internal organs^78^. Recent research suggests that innate immune memory influences the progression of these diseases by promoting proinflammatory cytokine release, metabolic reprogramming and epigenetic changes^71,78^.

In RA, proinflammatory cytokines such as TNF, IL-6 and IL-1β drive synovial hyperplasia, osteoclastogenesis and joint damage^79^. Monocytes from patients with RA are more susceptible to LPS-induced metabolic reprogramming leading to sustained inflammation and suggesting the occurrence of trained immunity^14,78,80^. β-glucans, known inducers of trained immunity, have been used in collagen-induced arthritis models and can trigger autoimmune arthritis in SKG models^81,82^. The injection of β-glucans increases clinical scores in mice by enhancing TNF secretion, autoantibody production and macrophage glycolysis^78,81^, whereas treatment with 2-deoxy-ᴅ-glucose, a nonmetabolizable glucose analog, reduces RA severity by inhibiting metabolic reprogramming^83^.

Earlier studies showed no enrichment of H3K4me3 in peripheral monocytes from patients with RA despite elevated serum levels^84^. However, recent findings show that CD14^+^ monocytes from patients with RA exhibit hyperinflammatory responses to LPS, increased cytokine production and metabolic reprogramming via the STAT3 pathway, leading to enhanced glycolysis^80^. RA-specific autoantibodies also induce trained immunity through metabolic reprogramming, with gene signatures enriched in synovial tissues^85^. Synovial fibroblasts play a key role in joint destruction in chronic RA inflammation, becoming primed by repeated challenges such as MSU or zymosan, leading to mTORC1-dependent metabolic reprogramming through the activation of complement C3 and its receptor C3a, ultimately driving a proinflammatory state^83^.

Bone marrow-mediated trained immunity is linked to inflammatory comorbidities, including the periodontitis–arthritis axis. In murine models, systemic inflammation triggered by periodontitis leads to epigenetic reprogramming of HSPCs, which results in prolonged myeloid cell production and increased inflammatory responses in arthritis. IL-1 signaling within HSPCs plays a critical role in the maladaptive training seen in periodontitis^86^. This form of systemic immune reprogramming may contribute to the progression and severity of comorbid autoimmune disorders, such as RA.

SLE is characterized by a diverse array of autoantibodies targeting various cellular components^87^. Recent studies highlight the role of monocytes/macrophages in SLE pathogenesis, showing increased TNF production and alterations in H3K4me3. Transcriptomic analyses of HSPCs from patients with SLE revealed an enhanced trained immunity signature^88^.

### Aging-related inflammation

As individuals age, the immune system undergoes substantial changes, increasing the susceptibility to diseases^89^. These age-related immune alterations, collectively termed immunosenescence, include tissue damage, low-grade systemic inflammation (inflammaging), reduced immune cell efficacy, suboptimal vaccination responses and heightened infection vulnerability^90^. Aging is associated with shifts in innate immune cell populations, notably the expansion of the nonclassical CD14^+^CD16^+^ monocyte subset, observed in healthy adults. This expansion is also evident in chronic conditions such as diabetes, atherosclerosis and CKD. Chronic low-grade inflammation can impair both innate and adaptive immune responses, potentially exacerbating tissue damage^91^. Notably, a recent study shows that BCG vaccination reduces systemic inflammation, and a lower baseline level of circulating inflammatory markers is linked to a stronger trained immunity response following vaccination in males^92^. By contrast, a recent randomized clinical trial involving BCG vaccination in the elderly found that BCG-induced trained immunity helps protect against new infections, particularly respiratory tract infections^34^. DAMPs, such as HMGB1 and oxLDL, contribute to chronic inflammation by persistently activating trained immunity and act as mediators of sterile inflammation in age-related conditions^90^. Consequently, the potential benefits of inducing trained immunity in elderly individuals with chronic inflammatory diseases, such as CVD, diabetes and CKD, should be thoroughly examined.

## Concluding remarks

Over the last 15 years, research has demonstrated that immune memory is an evolutionarily conserved characteristic in immune cells, challenging the long-standing belief that immune memory is exclusive to the adaptive immune system. Early studies emphasized the protective role of trained immunity against a range of pathogens, but it has since become clear that trained immunity can also have harmful effects in certain inflammatory contexts^14,93^. This duality mirrors adaptive immune memory, which can either aid immune responses or contribute to autoimmunity^94^. Trained immunity can sustain inflammation, leading to pathological conditions such as CVDs, autoimmune disorders and organ rejection. Moreover, chronic diseases have been linked to trained immunity induced by DAMPs or lifestyle-related triggers, both contributing to inflammatory processes. Excessive chronic inflammation induced by DAMPs or LAMPs may worsen inflammatory diseases, suggesting that therapies targeting trained immunity could benefit various inflammatory conditions.

**Fig. 4:**
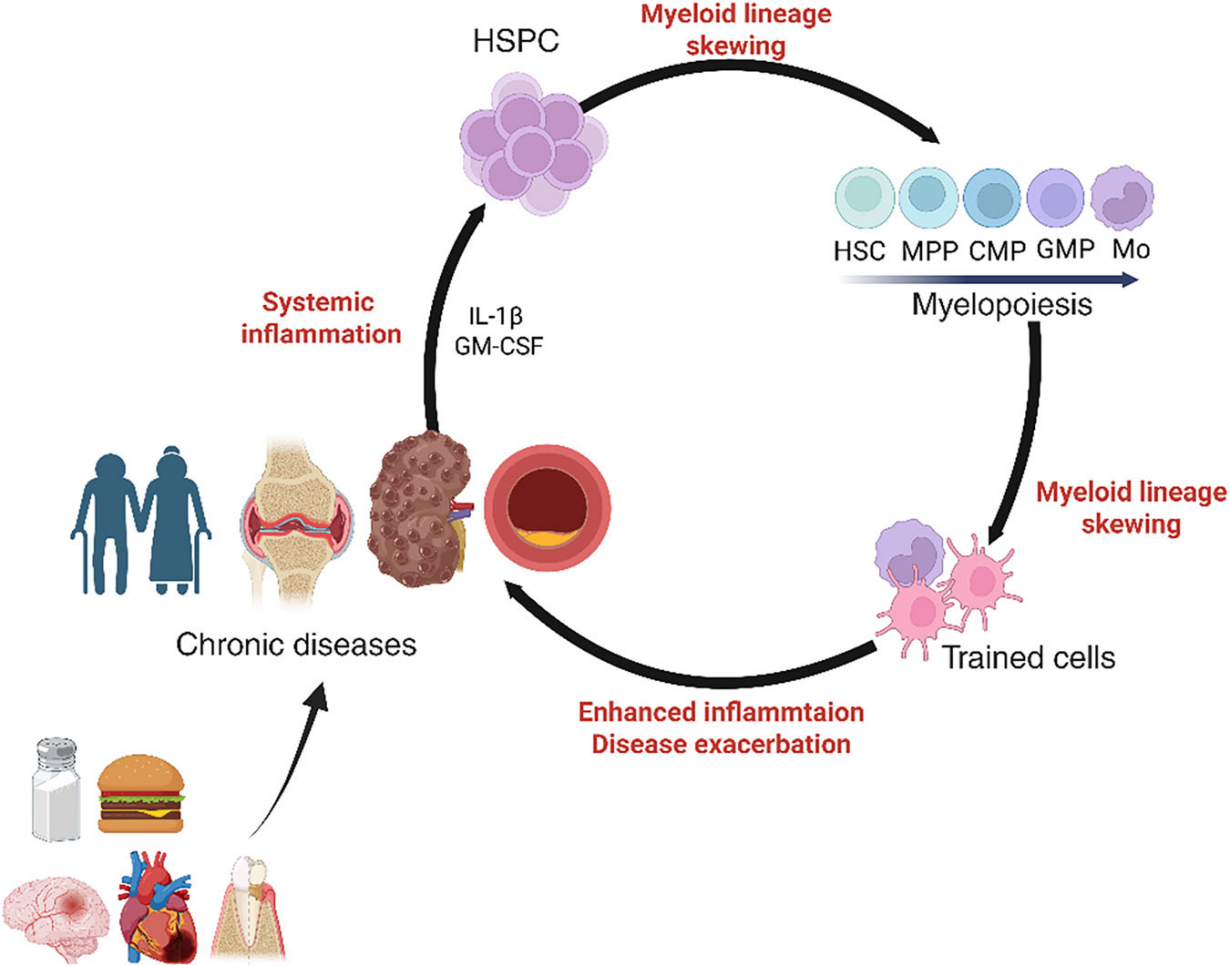
The interplay between trained immunity and chronic inflammatory diseases. This schematic illustrates the feed-forward loop in which both peripheral and central trained immunity drive the initiation and progression of chronic inflammatory diseases. In the early stages, endogenous and metabolic stimuli—such as oxLDL, WD, hyperlipidemia and hyperglycemia—induce a trained phenotype in innate immune cells (for example, monocytes and macrophages), leading to enhanced proinflammatory cytokine production and sustained low-grade systemic inflammation. Simultaneously, tissue injury or systemic insults (for example, heart failure, ischemic stroke and periodontitis) promote the expansion and myeloid-skewed differentiation of HSPCs in the bone marrow, reflecting central trained immunity. These interconnected processes reinforce one another, amplifying inflammation and contributing to the pathogenesis of chronic conditions such as CVD, CKD, rheumatic diseases and age-related inflammation (inflammaging). The figure was created using BioRender.com.

Recent evidence highlights a self-reinforcing loop between trained innate immunity and persistent inflammation, particularly in conditions such as atherosclerosis, SLE and sickle cell disease. Chronic exposure to inflammatory cytokines, DAMPs and LAMPs further amplifies this loop, worsening disease progression and promoting comorbidities^19,62,88,95–97^ (Fig. 4).

Much remains unclear about the mechanisms of DAMP- and LAMP-induced immunity compared with PAMP-induced trained immunity. Whereas metabolic reprogramming and epigenetic changes have been well characterized in trained immunity triggered by β-glucan or the BCG vaccine, the molecular pathways mediating the DAMP and LAMP response need further investigation. Moreover, appropriate animal models are needed to clarify the clinical relevance of these findings.

Given that trained immunity primarily involves metabolic rewiring and epigenetic reprogramming, these pathways are critical therapeutic targets. Inhibiting glycolysis or cholesterol metabolism, particularly mevalonate production, has been shown to reduce trained immunity^11,16,17,69^. Recently, myeloid-specific high-density lipoprotein nanotherapy has shown promise in regulating trained immunity^98^. Although the inhibition of trained immunity by high-density lipoprotein nanotherapy may benefit autoimmune diseases, the promotion of trained immunity has been shown to suppress tumor growth^99^. In addition, specific epigenetic modifications, such as acetylation or methylation of histone 3 at lysines 27 (H3K27) and 4 (H3K4), are key hallmarks of trained immunity. Inhibitors of histone modifications have been effective in suppressing trained immunity induced by both PAMPs and DAMPs^11^. Recent studies also suggest that innate immune memory following brain injury contributes to inflammatory cardiac dysfunction via IL-1β-mediated epigenetic changes^100^. Furthermore, the uremic toxin IS induces trained immunity through the AhR–AA pathway. Therefore, targeting IL-1β or the AA pathway could help mitigate trained immunity-related complications in conditions such as poststroke cardiac dysfunction and CKD^21^.

These findings emphasize the role of DAMP- and LAMP-mediated trained immunity in chronic inflammatory diseases. Future research to identify the sensors or signaling mechanisms central to DAMP- and LAMP-induced trained immunity may uncover new therapeutic targets. As DAMPs and LAMPs are increasingly relevant in aging and age-related inflammatory disorders, interest in this area is expected to grow.
